# Neuroprotective Effects of Qi Jing Wan and Its Active Ingredient Diosgenin Against Cognitive Impairment in Plateau Hypoxia

**DOI:** 10.3390/ph18050738

**Published:** 2025-05-17

**Authors:** Tiantian Xia, Ziqiao Yan, Pan Shen, Mingyang Chang, Nan Zhang, Yunan Zhang, Qi Chen, Rui Wang, Li Tong, Wei Zhou, Zhexin Ni, Yue Gao

**Affiliations:** 1 Department of Tranditional Chinese Medicine, Qinghai Unversity Medical College, Xining 810016, China; xiatianttx@163.com (T.X.); zn1445499093@163.com (N.Z.); qhtongli@126.com (L.T.); 2 Beijing Institute of Radiation Medicine, Beijing 100850, China; jason_yanziqiao@163.com (Z.Y.); spluto@foxmail.com (P.S.); chmingyang@163.com (M.C.); 20210931173@bucm.edu.cn (Y.Z.); cc313nn@163.com (Q.C.); zhouweisyl802@163.com (W.Z.); 3 Chinese PLA Medical School, Chinese People’s Liberation Army (PLA) General Hospital, Beijing 100036, China; 4 National Clinical Research Center for Chinese Medicine Acupuncture and Moxibustion, First Teaching Hospital of Tianjin University of Traditional Chinese Medicine, Tianjin 300193, China; 5 School of Life Sciences, Beijing University of Chinese Medicine, Beijing 102488, China; 6 General Hospital of Xinjiang Military Command, PLA, Urumqi 830000, China; urumqi@126.com; 7 State Key Laboratory of Kidney Diseases, Chinese PLA General Hospital, Beijing 100853, China

**Keywords:** high altitude, cognitive impairment, traditional herbal formula, diosgenin, brain organoids

## Abstract

**Background/Objectives**: High-altitude environments have a significant detrimental impact on the cognitive functions of the brain. Qi Jing Wan (QJW), a traditional herbal formula composed of *Angelica sinensis*, *Astragalus membranaceus*, and *Rhizoma Polygonati Odorati*, has demonstrated potential efficacy in treating cognitive disorders. However, its effects on cognitive dysfunction in plateau hypoxic environments remain unclear. **Methods**: In this study, acute and chronic plateau cognitive impairment mouse models were constructed to investigate the preventive and therapeutic effects of QJW and its significant active ingredient, diosgenin (Dio). Behavioral experiments were conducted to assess learning and memory in mice. Morphological changes in hippocampal neurons and synapses were assessed, and microglial activation and inflammatory factor levels were measured to evaluate brain damage. Potential active ingredients capable of crossing the blood–brain barrier were identified through chemical composition analysis and network database screening, followed by validation in animal and brain organoid experiments. Transcriptomics analysis, immunofluorescence staining, and molecular docking techniques were employed to explore the underlying mechanisms. **Results**: QJW significantly enhanced learning and memory abilities in plateau model mice, reduced structural damage to hippocampal neurons, restored NeuN expression, inhibited inflammatory factor levels and microglial activation, and improved hippocampal synaptic damage. Transcriptomics analysis revealed that Dio alleviated hypoxic brain damage and protected cognitive function by regulating the expression of PDE4C. **Conclusions**: These findings indicate that QJW and its significant active ingredient Dio effectively mitigate hypoxic brain injury and prevent cognitive impairment in high-altitude environments.

## 1. Introduction

The human brain is highly sensitive to hypoxia. In plateau regions, the unique hypobaric hypoxia environment leads to a significant reduction in systemic oxygen levels, which can readily trigger brain damage and negatively affect cognitive function [1,2]. Brief exposure to such environments often induces acute mountain sickness (AMS), characterized by symptoms such as headache and fatigue. This condition not only interferes with daily activities but also significantly impairs cognitive function. Specifically, short-term hypoxia exposure can lead to changes in cerebral blood flow and disorders in neurotransmitter metabolism, which in turn can weaken working and learning memory capacity, reduce attention span, and slow reaction times. The higher the altitude, the more pronounced the decline in attention and work capacity and the more severe the impairment of memory function [3,4]. Cognitive deficits become more severe with prolonged exposure to hypobaric hypoxia environments. Long-term exposure to high altitudes not only exacerbates cognitive dysfunction but also induces structural brain changes, including hippocampal volume reduction, cortical thickness decrease, neuronal atrophy and necrosis, and synaptic structural alterations. These structural changes are closely associated with cognitive dysfunction, suggesting that long-term plateau exposure may have significant detrimental effects on brain plasticity and neuroregenerative capacity [5]. With increasing economic prosperity and tourism development, the number of people migrating to plateau regions is gradually rising. This trend heightens the demand for effective health protection measures in plateau areas and underscores the critical need for the exploration of drugs to prevent and treat plateau cognitive impairment.

Numerous studies have demonstrated that components derived from medicinal plants, used either in the form of pharmaceuticals or nutraceuticals, can exert anti-cognitive disorder effects in humans [6,7]. Qi Jing Wan (QJW) consists of three food–medicine dual-use herbs: *Angelicae Sinensis Radix* (ASR), *Astragalus Membranaceus* (AM), and *Polygonati Rhizoma* (PR). These herbs possess the effects of nourishing and invigorating blood, replenishing qi, and elevating yang, as well as strengthening the spleen and benefiting the kidneys as per the traditional Chinese medicine theory. Pharmacological studies have demonstrated that certain active ingredients in ASR possess potent antioxidant and anti-inflammatory properties. These properties enable the ingredients to alleviate oxidative damage to nerve cells by scavenging free radicals and inhibiting oxidative stress. They also promote the elevated expression of neurotrophic factors, which supports neuronal survival and synaptic plasticity. Furthermore, these active ingredients inhibit the activity of acetylcholinesterase (AChE), thereby enhancing learning and memory abilities [8,9]. The primary active constituents of AM encompass saponins, flavonoids, and polysaccharides. These compounds work synergistically and exhibit a range of pharmacological effects, including anti-inflammatory, antioxidant, immunomodulatory, and neuroprotective pharmacological effects through synergistic interactions [10]. Additionally, AM may enhance cognitive function by reducing neuroinflammation, alleviating oxidative stress, inhibiting ferroptosis, and promoting neural regeneration and synaptic plasticity [11,12,13]. The chemical composition of PR is rich and diverse, primarily encompassing polysaccharides, saponins, flavonoids, amino acids, and volatile compounds. Notably, PR and its extracts have demonstrated significant potential in enhancing cognitive functions, particularly in improving learning and memory abilities, as supported by preclinical studies [14,15]. The aforementioned findings indicate that QJW holds considerable potential for the treatment of cognitive impairment. However, its specific role and the underlying mechanisms governing its action in plateau-induced cognitive impairment remain to be fully elucidated through further research.

In this study, we investigated the effects of QJW and its active ingredient, diosgenin (Dio), on preventing and treating plateau cognitive impairment using mouse models of acute and chronic plateau cognitive impairment. We also explored the specific mechanisms via a hypoxic brain organoid damage model. Initially, an acute plateau cognitive impairment model was established with a hypobaric oxygen chamber to assess QJW’s pharmacological effects and determine the optimal dose for the chronic study. Then, the chronic effects of QJW were examined by observing hippocampal neurons and synapses and by measuring the levels of microglia and inflammatory factors. The chemical composition of QJW was analyzed, with blood–brain barrier-crossing components identified for further evaluation. Transcriptomics revealed the gene PDE4C to be linked to Dio’s effects on hypoxic brain organoid damage, a finding validated using immunofluorescence and molecular docking.

## 2. Results

### 2.1. QJW Improves Acute Plateau Cognitive Impairment in Mice

**Figure 1:**
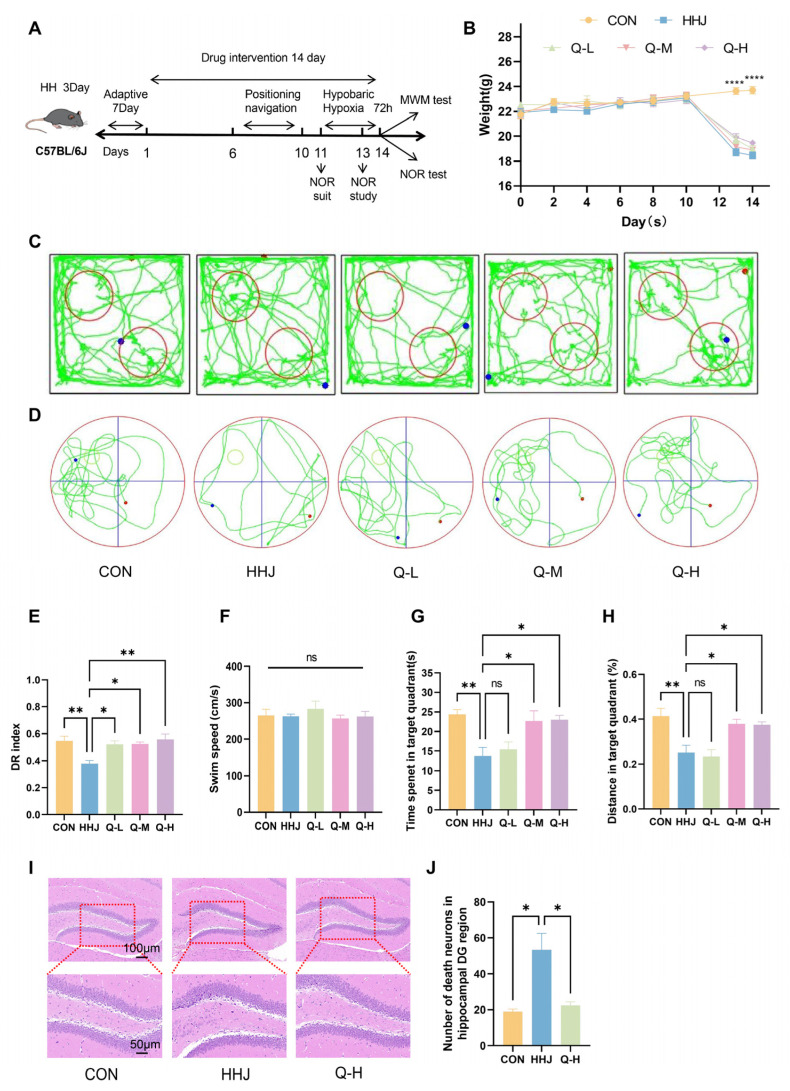
QJW improves acute plateau cognitive impairment in mice. (**A**) Flow chart of the experiment. (**B**) Weight change in mice (n = 8/group). (**C**) Motor trajectories of NOR mice. (**D**) Motor trajectories of MWM mice. (**E**) New object recognition test (NORT) (n = 6/group). (**F**) Swimming speed of mice during the MWM (n = 6/group). (**G**) Dwell time in the target quadrant (n = 6/group). (**H**) Percentage of swimming distance in the target quadrant (n = 6/group). (**I**) Hematoxylin and eosin (HE)-stained sections of the hippocampal dentate gyrus (DG) area. (**J**) Number of neuronal deaths in the hippocampal DG area (n = 3/group). Intergroup comparisons were performed using one-way ANOVA, followed by post hoc Tukey HSD testing. ns, *p* ≥ 0.05; *, *p* < 0.05; **, *p* < 0.01; ****, *p* < 0.0001.

Mice were exposed to a simulated 7000 m altitude for 72 h in a hypobaric hypoxia chamber to model acute plateau cognitive impairment. QJW treatment was administered 11 days prior to exposure, and its preventive effects were evaluated using the Novel Object Recognition Test (NOR) and Morris Water Maze Test (MWM) (Figure 1A). There was no significant difference in body weight between the groups of mice during the administration period. After entering the hypobaric oxygen chamber, the body weight of the model group and the administered group decreased rapidly and reached a minimum on the 14th day; there was a significant difference in body weights of all groups compared with that of the blank control (CON) group (*p* < 0.0001) (Figure 1B). Behavioral experiments (Figure 1C,D) revealed that, compared with the CON group, mice in the acute plateau model (HHJ) group exhibited a significant reduction in the proportion of time spent exploring novel objects in the NORT (*p* = 0.0044). QJW intervention significantly prevented acute plateau cognitive impairment compared to the HHJ group. Mice in the low- (Q-L), medium- (Q-M), and high-dose QJW (Q-H) groups showed significantly elevated proportions of time exploring novel objects in the Novel Object Recognition Test (NORT), with *p*-values of 0.0197 for Q-L, 0.0160 for Q-M, and 0.0024 for Q-H (Figure 1E). MWM revealed that, in the absence of significant differences in swimming speed (*p* > 0.05; Figure 1F), mice in the HHJ group exhibited significantly reduced exploration time (*p* = 0.0037; Figure 1G) and a significantly lower percentage of exploration distance in the target quadrant (*p* = 0.0019; Figure 1H) compared with the CON group. In contrast, mice receiving QJW demonstrated significantly increased exploration time in the target quadrant for Q-M (*p* = 0.0176) and Q-H (*p* = 0.0133) but not for Q-L (*p* = 0.9718) (Figure 1G). Similarly, the proportion of exploration distance in the target quadrant was significantly higher for Q-M (*p* = 0.0184) and Q-H (*p* = 0.0242) but not for Q-L (*p* = 0.9938) (Figure 1H). The results indicated that QJW had a significant anti-acute plateau cognitive impairment effect, with the best effect at high doses. Further exploration of mouse hippocampal HE-stained images revealed (Figure 1I) that the HHJ group exhibited a significantly higher number of necrotic neurons in the hippocampus compared with the CON group (*p* = 0.0106). Additionally, high-dose QJW (Q-H) alleviated neuronal cell consolidation necrosis caused by hypobaric hypoxia (HH) compared with the HHJ group (*p* = 0.0169; Figure 1J). The above results suggest that QJW should be further investigated as a potential anti-plateau cognitive impairment drug.

### 2.2. QJW Improves Chronic Plateau Cognitive Impairment in Mice

**Figure 2:**
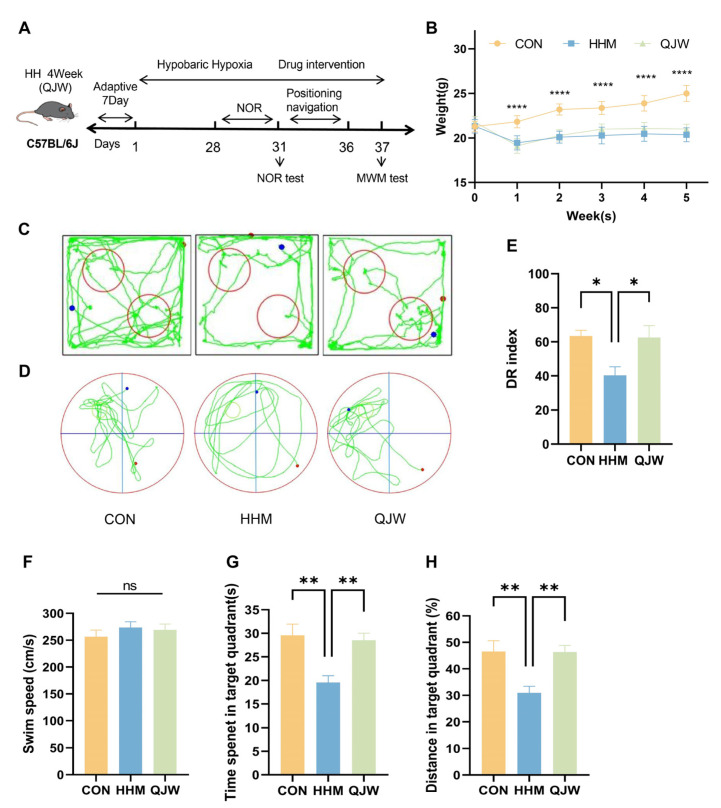
QJW improves chronic plateau cognitive impairment in mice. (**A**) Flow chart of the experiment. (**B**) Weight change in mice (n = 8/group). (**C**) Motor trajectories of NOR mice. (**D**) Motor trajectories of MWM mice. (**E**) New object recognition test (NORT) (n = 6/group). (**F**) Swimming speed of mice during the MWM (n = 6/group). (**G**) Dwell time in the target quadrant (n = 6/group). (**H**) Percentage of swimming distance in the target quadrant (n = 6/group). Intergroup comparisons were performed using one-way ANOVA, followed by post hoc Tukey HSD testing. ns, *p* ≥ 0.05; *, *p* < 0.05; **, *p* < 0.01; ****, *p* < 0.0001.

To explore QJW’s effects on chronic plateau cognitive impairment, mice were exposed to a simulated 6000 m altitude for 4 weeks in a hypobaric hypoxic chamber. The preventive and therapeutic effects of QJW were evaluated via behavioral experiments (Figure 2A). Compared with the CON group, mice in both the chronic plateau model (HHM) and the chronic plateau QJW group showed a significant decrease in body weight one week after entering the chamber (*p* < 0.0001), followed by a gradual increase over the subsequent three weeks. There was no significant difference in body weight between the HHM and QJW groups (Figure 2B). Moreover, hematoxylin and eosin (HE) staining of the liver (Figure S1D) revealed that QJW ameliorated the structural disorganization of the central hepatic vein, hepatocyte swelling, and vacuolar degeneration in plateau model mice. These findings indicate that QJW exerts a protective effect without causing additional damage. Collectively, these results, in conjunction with the observed body weight changes, suggest that QJW has a favorable safety profile. Behavioral experiments in mice revealed (Figure 2C,D) that, compared with the CON group, mice in the HHM group exhibited a significant reduction in the proportion of time spent exploring novel objects in the NORT (*p* = 0.0208). Additionally, compared with the HHM group, the QJW intervention significantly alleviated chronic plateau cognitive impairment, with mice receiving QJW showing a significant increase in the proportion of time spent exploring novel objects in the NORT (*p* = 0.0264) (Figure 2E). The MWM revealed that, in the absence of significant differences in swimming speed (*p* > 0.05; Figure 2F), mice in the HHM group exhibited significantly reduced exploration time in the target quadrant (*p* = 0.0040) and a significantly lower percentage of exploration distance (*p* = 0.0073) compared with the CON group. Conversely, mice in the QJW group demonstrated significantly increased exploration time in the target quadrant (*p* = 0.0089) and a significantly higher percentage of exploration distance (*p* = 0.0080) (Figure 2G,H). These results demonstrate that QJW has a significant protective effect against chronic plateau cognitive impairment.

### 2.3. QJW Alleviates Hippocampal Neuronal and Synaptic Damage in Mice Under Hypobaric Hypoxia

**Figure 3:**
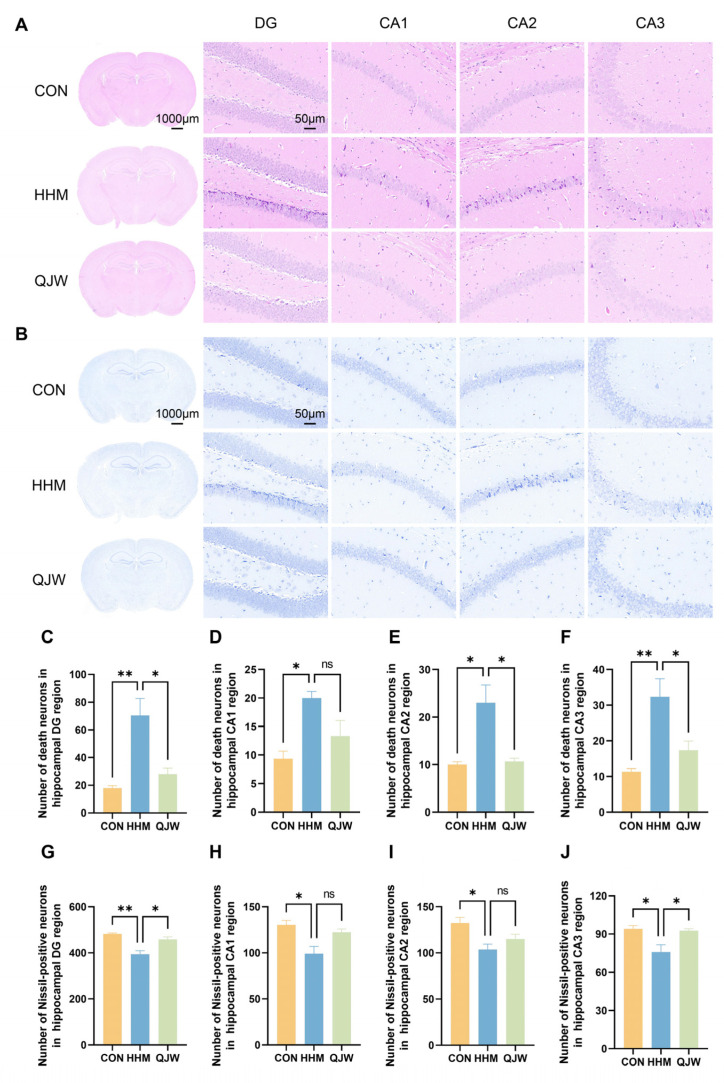
QJW alleviates hippocampal neuronal damage in mice with chronic plateau cognitive impairment. (**A**) Representative images of HE staining in the mouse hippocampus. (**B**) Representative images of Nissl staining in the mouse hippocampus. (**C**) Number of neuronal deaths in the hippocampal DG region. (**D**) Number of neuronal deaths in the hippocampal CA1 region. (**E**) Number of neuronal deaths in the hippocampal CA2 region. (**F**) Number of neuronal deaths in the hippocampal CA3 region. (**G**) Number of Nissl-positive neurons in the hippocampal DG region. (**H**) Number of Nissl-positive neurons in the hippocampal CA1 region. (**I**) Number of Nissl-positive neurons in the hippocampal CA2 region. (**J**) Number of Nissl-positive neurons in the hippocampal CA3 region. Intergroup comparisons were performed using one-way ANOVA, followed by post hoc Tukey HSD testing. ns, *p* ≥ 0.05; *, *p* < 0.05; **, *p* < 0.01 (n = 3/group).

**Figure 4:**
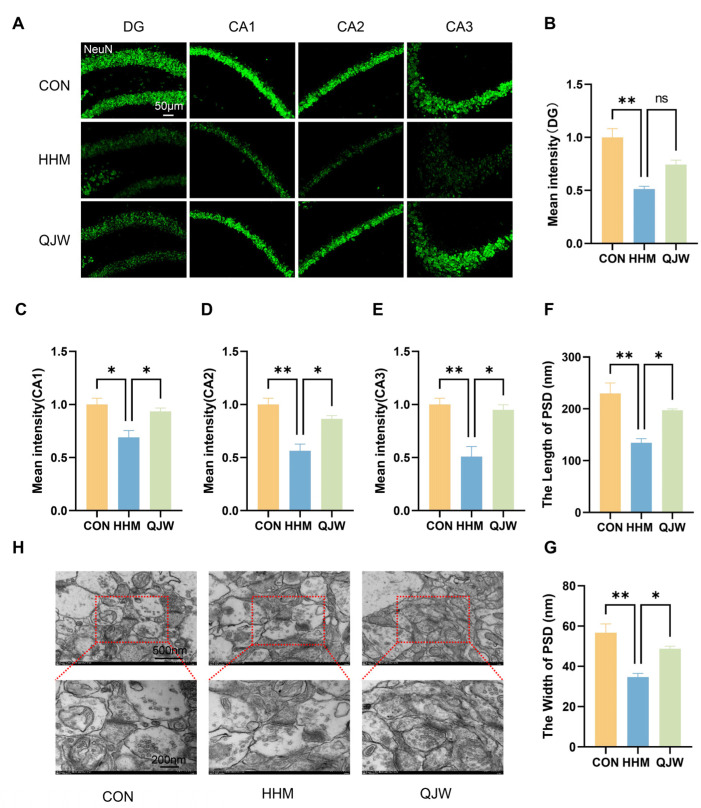
QJW protects against HH-induced neuronal and synaptic damage in the mouse hippocampus. (**A**) Representative images of NeuN immunofluorescence staining. (**B**) Average fluorescence intensity of NeuN in the hippocampal DG region. (**C**) Average fluorescence intensity of NeuN in the hippocampal CA1 region. (**D**) Average fluorescence intensity of NeuN in the hippocampal CA2 region. (**E**) Average fluorescence intensity of NeuN in the hippocampal CA3 region. (**F**) Length of hippocampal synaptic PSD. (**G**) Width of hippocampal synaptic PSD. (**H**) Representative images of hippocampal synapses. Intergroup comparisons were performed using one-way ANOVA, followed by post hoc Tukey HSD testing. ns, *p* ≥ 0.05; *, *p* < 0.05; **, *p* < 0.01 (n = 3/group).

The hippocampus in the brain is a critical structural region for maintaining cognitive function. Hematoxylin and eosin (HE) staining was used to assess morphological changes in the mouse hippocampus (Figure 3A). Compared with the CON group, the number of sequestered necrotic cells in the hippocampal DG, CA1, CA2, and CA3 regions of mice in the HHM group was significantly increased (Figure 3C–F). After QJW administration, the number of necrotic neurons in the DG (*p* = 0.0186; Figure 3C), CA2 (*p* = 0.0190; Figure 3E), and CA3 (*p* = 0.0427; Figure 3F) regions was significantly reduced. Nissl (Ni) staining showed that exposure to hypoxia resulted in a decrease in Ni-positive cells (Figure 3B,G–J). After QJW administration, the number of Ni-positive cells in the hippocampal DG (*p* = 0.0107; Figure 3G) and CA3 (*p* = 0.0441; Figure 3J) regions was significantly increased. NeuN immunofluorescence staining of the hippocampal region revealed that NeuN expression in all regions of the hippocampus of mice in the HHM group was significantly decreased compared with that in the CON group (Figure 4B–E). After QJW administration, protein expression in the CA1 (*p* = 0.0400; Figure 4C), CA2 (*p* = 0.0171; Figure 4D), and CA3 regions (*p* = 0.0106; Figure 4E) was significantly restored. These results indicated that QJW had a protective effect on hippocampal neurons in mice with chronic cognitive impairment induced by long-term hypoxia. Ultrastructural observation of hippocampal synapses showed that QJW significantly restored the hypoxia-induced reduction in the length (*p* = 0.0301; Figure 4F) and width (*p* = 0.0268; Figure 4G) of hippocampal synaptic PSD in mice (Figure 4H).

### 2.4. QJW Inhibits Long-Term Hypobaric-Hypoxia-Induced Neuroinflammatory Responses

**Figure 5:**
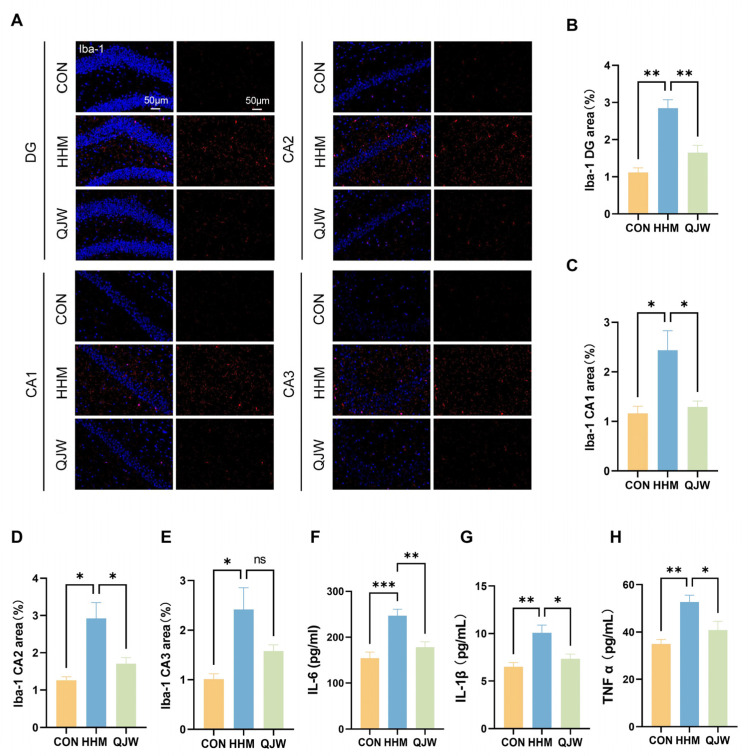
QJW inhibits HH-induced neuroinflammatory responses. (**A**) Representative images of Iba-1 immunofluorescence staining. (**B**) Fluorescence intensity volume ratio in the hippocampal DG region. (**C**) Fluorescence intensity volume ratio of Iba-1 in the hippocampal CA1 region. (**D**) Fluorescence intensity volume ratio of Iba-1 in the hippocampal CA2 region. (**E**) Fluorescence intensity volume ratio of Iba-1 in the hippocampal CA3 region (n = 3/group). (**F**) Serum IL-6 levels. (**G**) Serum IL-1β levels. (**H**) Serum TNF-α levels (n = 6/group). Intergroup comparisons were performed using one-way ANOVA, followed by post hoc Tukey HSD testing. ns, *p* ≥ 0.05; *, *p* < 0.05; **, *p* < 0.01; ***, *p* < 0.001.

Cognitive impairment is often accompanied by elevated inflammatory markers in vivo, and aberrant activation of the brain’s immune microenvironment is thought to be an important driver of cognitive impairment. To investigate whether hypobaric-hypoxia-induced chronic cognitive impairment is associated with neuroinflammation, we examined the immunofluorescence expression of hippocampal Iba-1 and serum levels of inflammatory factors in mice. Immunofluorescence results showed that microglia in the hippocampus of mice in the HHM group were significantly activated compared with those in the CON group (Figure 5A). The Iba-1 fluorescence intensity volume ratios in the hippocampus in the DG area (*p* = 0.0092; Figure 5B), the CA1 area (*p* = 0.0429; Figure 5C), and the CA2 area (*p* = 0.0423; Figure 5D) were significantly decreased after QJW treatment. The results of the serum inflammatory factor assay showed that QJW significantly reduced the long-term hypobaric-hypoxia-induced elevation of IL-6 (*p* = 0.0054; Figure 5F), IL-1β (*p* = 0.0138; Figure 5G), and TNF-α (*p* = 0.0307; Figure 5H) levels. These findings suggest that QJW may reduce the levels of inflammatory factors in vivo by inhibiting the activation of microglial cells, thereby alleviating long-term hypobaric-hypoxia-induced neuroinflammation and attenuating chronic plateau cognitive impairment.

### 2.5. UPLC-MS/MS Assay for QJW and Screening of Potential Brain-Entry Components

**Figure 6:**
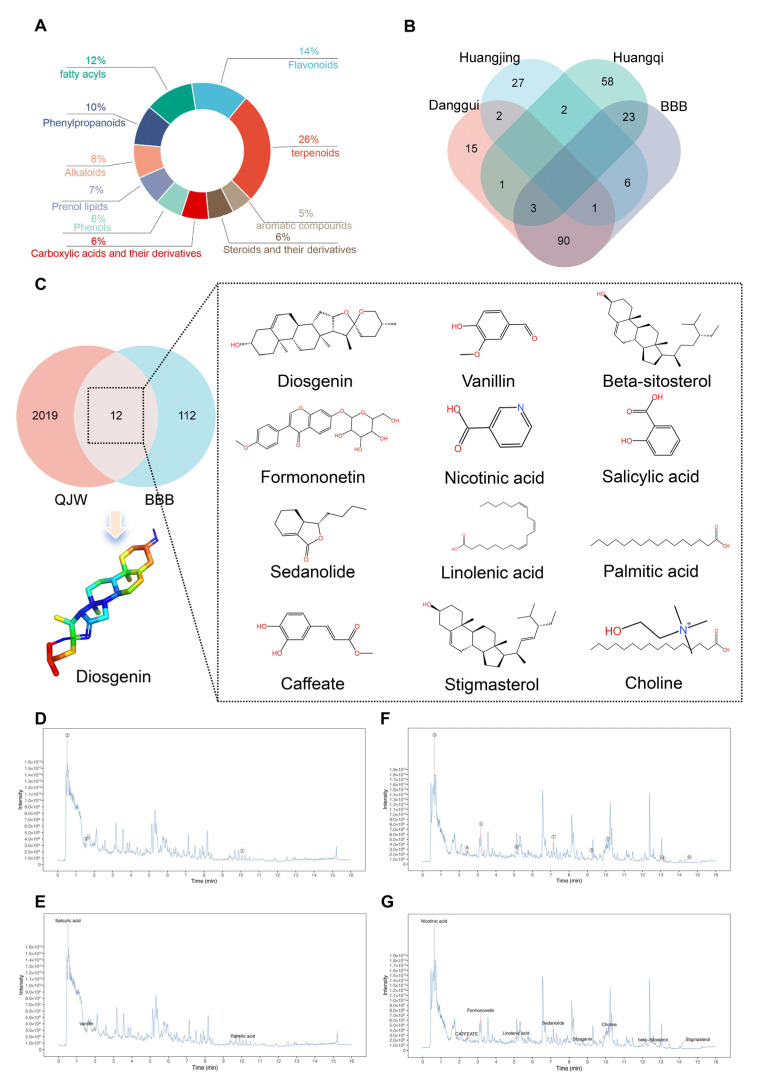
UPLC-MS/MS detection of QJW and screening of potential brain-entry components. (**A**) Top ten ingredient species in QJW. (**B**) The components of ASR (Danggui), PR (Huangjing), and AM (Huangqi) were intersected with those of the BBB to identify overlapping constituents. (**C**) The chemical structural formulas of the 12 components in QJW that have the potential to pass through the blood–brain barrier. (**D**,**E**) Positive ion mass spectra of 12 components in QJW that potentially cross the blood–brain barrier. (**F**,**G**) Negative ion mass spectra of 12 components in QJW that potentially cross the blood–brain barrier.

To identify the active ingredients of QJW that may alleviate cognitive impairment in plateau environments, UPLC-MS/MS analysis was conducted on the lyophilized powder of QJW. The results indicated that the top five chemical constituent categories in QJW were terpenoids, flavonoids, fatty acyls, phenylpropanoids, and alkaloids (Figure 6A). Further investigation using the TCMSP database revealed a total of 123 components from QJW that could theoretically cross the blood–brain barrier (BBB ≥ −0.03). Specifically, 94, 7, and 26 components were identified in Angelica sinensis, Polygonati Rhizoma, and Astragalus membranaceus, respectively (Figure 6B). By comparing the TCMSP database entries with the chemical compositions of QJW, 12 potential brain-entry components were identified (Supplementary Table S1); their structural formulas are presented in Figure 6C. The positive ion mass spectra (Figure 6D,E) and negative ion mass spectra (Figure 6F,G) of QJW are also shown. Through screening for drug-like properties (DL ≥ 0.18) and oral bioavailability (OB ≥ 30%), diosgenin (Dio) was identified as an active ingredient with the highest oral bioavailability and the potential for improving cognitive impairment in plateau environments and was thus selected for further study.

### 2.6. Dio Improves Plateau Cognitive Impairment in Mice

**Figure 7:**
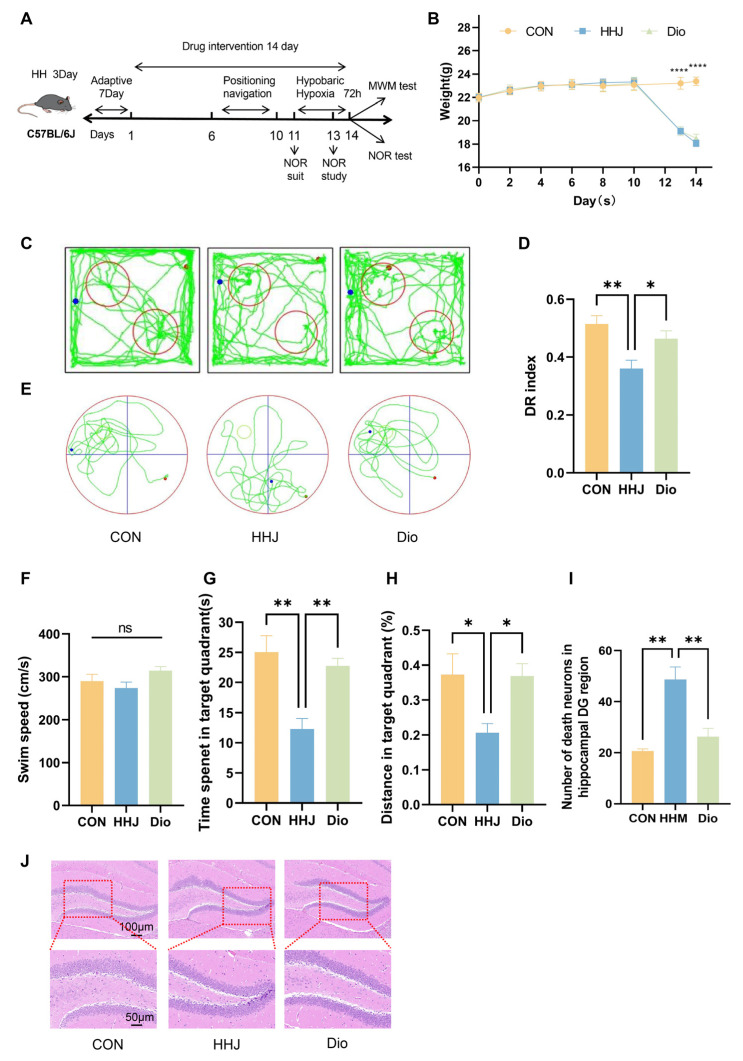
Dio improves acute plateau cognitive impairment in mice. (**A**) Flow chart of the experiment. (**B**) Weight change in mice (n = 8/group). (**C**) Motor trajectories of NOR mice. (**D**) New object recognition test (NORT) (n = 6/group). (**E**) Motor trajectories of MWM mice. (**F**) Swimming speed during the MWM (n = 6/group). (**G**) Dwell time in the target quadrant (n = 6/group). (**H**) Percentage of swimming distance in the target quadrant (n = 6/group). (**I**) Number of neuronal deaths in the hippocampal DG area (n = 3/group). (**J**) HE-stained sections of the hippocampal DG area (n = 3/group). Intergroup comparisons were performed using one-way ANOVA, followed by post hoc Tukey HSD testing. ns, *p* ≥ 0.05; *, *p* < 0.05; **, *p* < 0.01; ****, *p* < 0.0001.

To assess Dio’s protective effects against acute plateau cognitive impairment, mice were exposed to a simulated 7000 m altitude for 72 h using a hypobaric hypoxic chamber. Dio treatment began 11 days prior to exposure, and its preventive effects were evaluated via the NOR and MWM (Figure 7A). The changes in body weight between groups of mice during administration were similar to the results for QJW administration. Compared with the CON group, the body weights of all groups of mice showed significant differences (*p* < 0.0001) after entering the chamber (Figure 7B). Behavioral tests in mice revealed (Figure 7C,E) that, compared with the CON group, mice in the HHJ group exhibited a significant reduction in the proportion of time spent exploring novel objects in the NORT (*p* = 0.0039). Additionally, compared with the HHJ group, Dio intervention significantly prevented cognitive impairments in the plateau environment, with mice receiving Dio showing a significant increase in the proportion of time spent exploring novel objects in the NORT (*p* = 0.0483) (Figure 7D). The MWM revealed that, in the absence of significant differences in swimming speed (*p* > 0.05; Figure 7F), mice in the HHJ group exhibited significantly reduced exploration time in the target quadrant (*p* = 0.0010) and a significantly lower percentage of exploration distance (*p* = 0.0378) compared with the CON group. Compared with the HHJ group, mice in the Dio group exhibited a significantly increased exploration time in the target quadrant (*p* = 0.0054) and a significantly higher proportion of exploration distance (*p* = 0.0435) (Figure 7G,H). Further examination of mouse hippocampal HE-stained images revealed that the HHJ group exhibited a significantly higher number of necrotic neurons in the hippocampus compared with the CON group (*p* = 0.0029). Additionally, Dio administration prevented hypoxia-induced neuronal necrosis compared to the HHJ group (*p* = 0.0090; Figure 7J). These results demonstrate that Dio has a significant preventive effect against acute plateau cognitive impairment.

### 2.7. Dio Ameliorates Hypoxic Brain Organoid Damage

**Figure 8:**
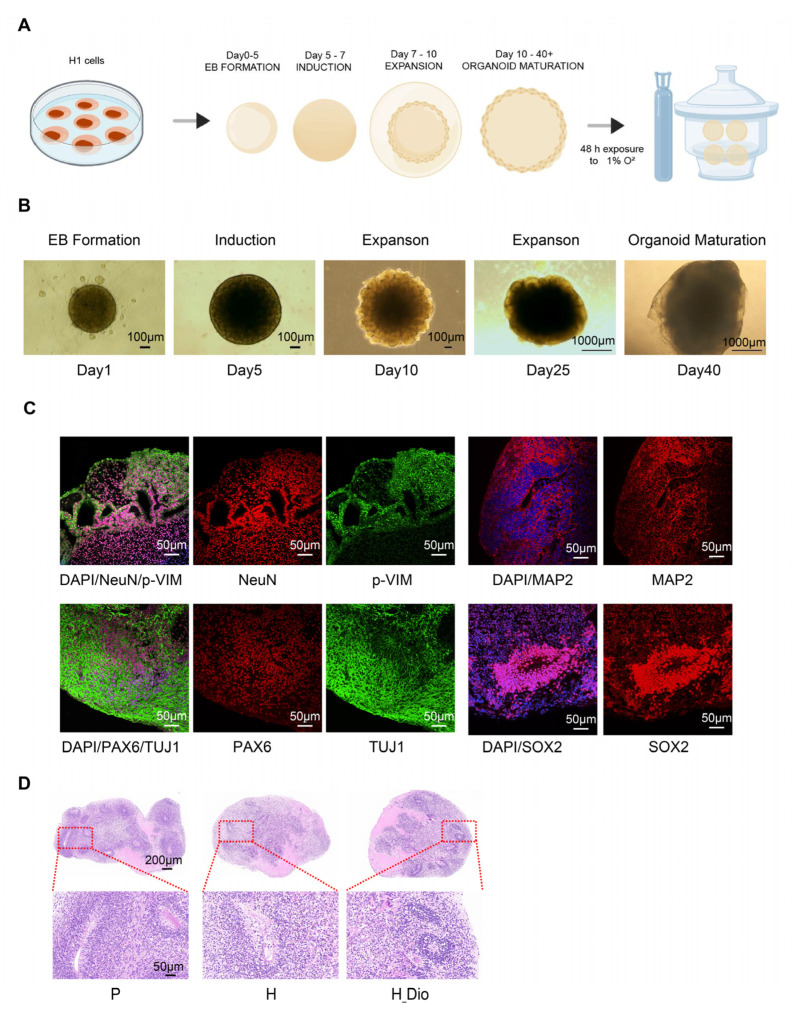
Dio ameliorates hypoxic brain organoid damage. (**A**) Flow chart depicting the experimental procedure for inducing brain organoids from H1 cells. (**B**) Representative images depicting the growth of brain organoids at various developmental stages. (**C**) Representative immunofluorescence images of NeuN, p-VIM, PAX6, TUJ1, MAP2, and SOX2 proteins within brain organoids are presented. (**D**) Representative images of the HE staining of brain organoids.

To explore the protective effect of Dio on hypoxic brain organoids, H1 cells were cultured under embryonic stem cell conditions and grown in a colony-like manner. After the induction of differentiation, they gradually formed embryoid bodies and neural ectoderm. Microscopic observations during growth showed that brain organoid tissues increased in size and gradually differentiated into distinct brain region structures. Mature organoids were identified and cultured in a hypoxic chamber (1% O2) for 48 h to construct a model of hypoxic injury (Figure 8A). After 40 days of culture, the human brain organoids matured (Figure 8B), and immunofluorescence revealed dynamic co-expression of multi-stage neurodevelopmental markers within the organoid tissues: neural stem cell marker SOX2, cortical developmental regulator PAX6, neuron-specific structural proteins MAP2 and p-VIM, early neuronal marker TUJ1, and mature neuronal nuclear protein NeuN (Figure 8C). These results indicated a successful brain organoid culture. HE staining of brain organoids after the Dio intervention showed that Dio restored the number of neural rosettes in hypoxia-injured organoids and significantly increased the number of neurons surrounding the rosettes (Figure 8D). These findings suggest that Dio can alleviate hypoxia-induced brain organoid damage.

### 2.8. Dio Alleviates Hypoxia-Induced Damage to Brain Organoids by Regulating PDE4C

**Figure 9:**
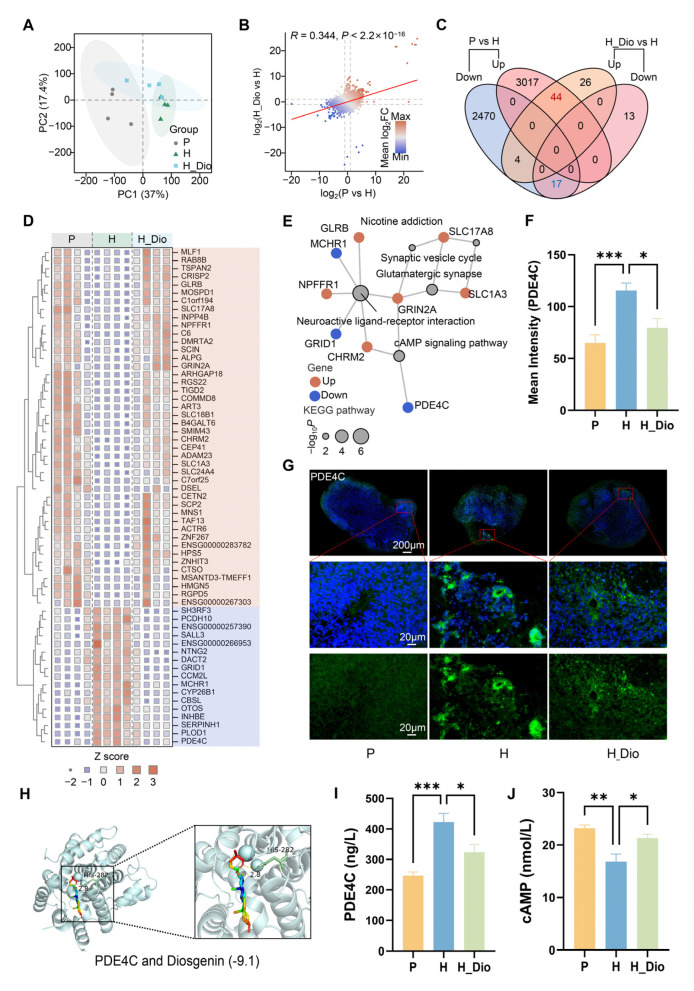
Dio alleviates hypoxia-induced organoid damage in the human brain by regulating key genes. (**A**) PCA. (**B**) Differential gene expression analysis between P vs. H and H_Dio vs. H groups. (**C**) Venn diagrams of differential gene intersections between P vs. H and H_Dio vs. H groups. (**D**) Heatmap of differential gene expression and clustering in the three groups. (**E**) KEGG pathway enrichment analysis of differential genes (n = 4/group). (**F**) Mean fluorescence intensity of PDE4C (n = 9/group). (**G**) Representative images of PDE4C immunofluorescence. (**H**) Molecular docking of PDE4C with Dio. (**I**) PDE4C protein content in brain-like organs. (**J**) Brain organ cAMP protein content (n = 6/group). Intergroup comparisons were performed using one-way ANOVA, followed by post hoc Tukey HSD testing. *, *p* < 0.05; **, *p* < 0.01; ***, *p* < 0.001.

To investigate the effect of Dio on gene expression changes in brain organoids after hypoxia, transcriptome sequencing was performed on different groups of brain organoids in this study. The transcriptome data were first analyzed by dimensionality reduction using principal component analysis (PCA) (Figure 9A). The results showed a clear separation between the normal control group (P) and the hypoxic group (H), while the hypoxic group treated with Dio (H_Dio) was located between these two groups. This suggests that Dio has a protective effect on the overall transcriptional changes induced by hypoxia in human brain organoids. Differential expression analyses were then performed between the P vs. H and H_Dio vs. H groups. The correlation of log2-transformed fold change between these two comparisons showed a positive correlation (R = 0.344) (Figure 9B), which is consistent with the results obtained with PCA. Functional analyses of differentially expressed genes (DEGs) between the P vs. H and H_Dio vs. H groups showed that, in the P vs. H groups, the up-regulated DEGs were enriched for functions related to DNA damage repair, whereas the down-regulated DEGs were mainly enriched for hypoxia-responsive pathways, such as the hypoxia response and the HIF-1 signaling pathway (Figure S1A). In contrast, up- and down-regulated DEGs in the H_Dio vs. H groups were more likely to be involved in neurotransmitter-related processes, including neuroactive ligand–receptor interactions, glutamatergic synapses, and neurotransmitter transport (Figure S1B).

Further screening of DEGs with consistent expression trends in the P vs. H and H_Dio vs. H groups identified 44 genes that were up-regulated and 17 genes that were down-regulated in the comparison of the two groups (Figure 9C). These 44 up-regulated genes showed specific down-regulation in group H, while the 17 down-regulated genes showed specific up-regulation in group H (Figure 9D). KEGG pathway enrichment analysis of these DEGs (Figure 9E) showed that pathways such as neuroactive ligand–receptor interactions, glutamatergic synapses, the cAMP signaling pathway, nicotine addiction, and the synaptic vesicle cycle were enriched. This suggests that Dio can protect human brain organoids from hypoxic injury by regulating key biological pathways that are essential for their normal function.

Among these enriched pathways, the cAMP signaling pathway, in which PDE4C is involved, plays an important role in regulating neuronal survival, axonal and dendritic growth, and synaptic plasticity in neural growth and development. PDE4C expression was significantly down-regulated in both comparative groups, P vs. H and H_Dio vs. H. Specifically, PDE4C expression was up-regulated after hypoxia exposure but recovered after Dio treatment. Importantly, PDE4C had the largest mean fold difference in both comparison groups (Figure S1C), suggesting that it may play a crucial role in hypoxic brain organoid damage.

Further validation of this gene using immunofluorescence staining (Figure 9G) showed that the Dio intervention significantly restored the hypoxia-induced abnormally elevated mean fluorescence intensity of PDE4C (*p* = 0.0080; Figure 9F). Molecular docking results showed that Dio had a strong affinity for PDE4C, with a binding energy of -9.1 kcal/mol (Figure 9H). The enzyme-linked immunosorbent assay (ELISA) results revealed that the protein expression of PDE4C was significantly higher in group H compared to group P (*p* = 0.0002) and significantly lower in group H_Dio compared to group H (*p* = 0.0225) (Figure 9I). Additionally, the protein expression of cAMP was significantly lower in group H compared to group P (*p* = 0.0010) and significantly higher in group H_Dio compared to group H (*p* = 0.0161) (Figure 9J).These findings suggest that Dio is closely associated with the expression levels of PDE4C and cAMP.

## 3. Discussion

Exposure to hypobaric hypoxia environments elicits a range of uncomfortable symptoms in humans. The brain is highly sensitive to hypoxia and is prone to hypoxic injury, resulting in cognitive decline [5]. This study established a hypobaric hypoxia mouse model and evaluated QJW’s protective effects through behavioral tests, histopathology, hippocampal ultrastructure analysis, and inflammation assays. Dio, identified as a blood–brain barrier-penetrating compound, was further studied for its mechanism in alleviating hypoxic neurological injury using both animal models and brain organoids. In summary, our study explored the beneficial effects of QJW on plateau-induced cognitive impairment and provided a foundation for its further research.

The Noval Object Recognition Test (NOR) and Morris Water Maze Test (MWM) are widely used to evaluate learning and memory abilities in rodents [16,17]. During learning, hippocampal neurons encode and store information via synaptic plasticity mechanisms. Behavioral experiments revealed that QJW administration can effectively ameliorate the learning and spatial memory deficits induced by hypobaric hypoxia environments in plateau model mice. The hippocampus is a critical region for memory and spatial cognition. Neurons, as the fundamental units of the nervous system, are essential for maintaining cognitive functions. In learning and memory processes, hippocampal neurons are responsible for receiving, processing, and transmitting information, and their functional integrity directly impacts cognitive performance [18]. Histological findings confirmed the neuroprotective and repair-promoting effects of QJW in plateau model mice.

The development of cognitive dysfunction is closely associated with abnormal levels of systemic inflammation [19]. In this study, we observed that QJW administration mitigated the increased levels of inflammatory factors induced by the low-pressure hypoxic environment. Elevated inflammatory markers in vivo often indicate the activation of microglia. Abnormal activation of the brain’s immune microenvironment is considered a key driver of cognitive impairment. Persistent activation of astrocytes and microglia not only disrupts normal neuronal communication but also alters the blood–brain barrier permeability, leading to abnormal infiltration of peripheral immune cells and creating a persistent neuroinflammatory microenvironment that further exacerbates neuroinflammation [20,21]. Iba-1 immunofluorescence staining indicated that QJW may mitigate neuroinflammation by inhibiting microglial activation, thereby improving cognitive impairment.

Synapses are critical sites for information transfer and functional activity within the interneuronal system, and their coordinated expression of synaptic plasticity underpins learning and memory processes, being closely related to cognitive function [22,23]. Synaptic plasticity refers to the capacity of synaptic efficacy to change in response to neuronal activity, a property that constitutes the neurological basis of learning and memory [24]. Damage to hippocampal neurons and synapses leads to severe cognitive impairment [25,26]. Inhibition of synaptic plasticity is closely associated with microglial activation and the release of inflammatory factors [27]. These findings suggest that cognitive impairment is associated with various factors, such as neuroinflammation and synaptic damage; however, the specific gene regulatory pathways remain unclear. Microglia, as core cells of the central nervous system, are closely associated with inflammatory signaling networks through their dynamic interactions with synapses. Under normal physiological conditions, these cells participate in the dynamic regulation of the synaptic structure with the help of low levels of inflammatory molecules. However, under pathological conditions, excessive inflammatory signaling becomes a significant pathological basis for the development of neurodegenerative and psychiatric diseases [28]. Studies have shown that microglia are involved in synaptic pruning processes via the classical complement pathway. For example, in Alzheimer’s disease (AD), the aberrant accumulation of β-amyloid specifically activates intracellular inflammasomes, contributing to the overproduction of the pro-inflammatory factors IL-1β and TNF-α, which impair synaptic signaling efficiency [29]. In this study, we found that QJW administration reduced the elevated inflammation levels induced by exposure to the hypobaric hypoxia environment of the plateau, attenuated the abnormal synaptic modifications induced by microglial activation, and protected hippocampal neurons and synaptic structures, findings that are consistent with those of a previously reported study [30].

UPLC-MS/MS was employed to identify and analyze bioactive compounds within QJW and further investigate the active components that exert preventive effects [31]. In order to elucidate the specific components of QJW that play a role in preventing and treating cognitive impairment in plateau environments, the chemical composition of QJW lyophilized powder was examined in this study. By comparing with TCMSP, a database of traditional Chinese medicines, we screened out Dio, a potential active ingredient that passes through the blood–brain barrier, and verified the screened Dio in animal experiments in the acute plateau model mice. Behavioral experiments and histological analyses demonstrate that Dio effectively prevents hypoxia-induced neuronal damage and protects cognitive function. This study demonstrates the preventative and therapeutic effects of QJW and its potential brain-penetrating constituent, Dio, on high-altitude cognitive impairment. Future research could further investigate the synergistic effects of QJW and Dio, evaluating their potential to prevent and treat cognitive deficits associated with high-altitude exposure.

Brain organoids are three-dimensional cellular models differentiated from induced pluripotent stem cells or embryonic stem cells that are capable of replicating the developmental process and some functional features of the human brain [32]. In recent years, researchers have increasingly used brain organoids in neurodegenerative disease research. These organoids can effectively reproduce the core pathological features of many cognitive disorders, including AD models and neuroinflammation, as well as other neurodegenerative pathways [33,34]. For example, in AD models, brain organoids can recapitulate the formation of Aβ amyloid plaques and tau protein neurofibrillary tangles, thereby revealing the potential impact of these pathological changes on neuronal and synaptic functions [35]. Brain organoids have thus opened new avenues for investigating the pathogenesis of cognitive disorders. For instance, by analyzing the gene expression and epigenetic changes in organoids, researchers have gained insights into the molecular basis of cognitive disorders [36]. Other studies have demonstrated that hypoxia causes significant damage to neurons in brain organoids [37,38]. This finding is particularly relevant for studying the combination of plateau hypoxic brain damage and brain organoids. In this study, we used mature brain organoids and referred to the hypoxic brain injury conditions described in previous studies to establish a hypoxic injury model in them [36]. We then pharmacologically intervened using Dio and conducted transcriptome analyses to explore its mechanism of action. The PCA results showed a significant separation between groups, indicating that Dio exerted a protective effect on hypoxia-induced transcriptional changes in human brain organoids.

GO enrichment is associated with functions related to DNA damage repair, hypoxia response pathways, and neurotransmitter-related processes. Hypoxia may impede the proliferation and differentiation of neuronal cells, influencing the growth and development of the nervous system, the maturation of brain structure and function, and consequently, cognitive function [39]. Hypoxia induces an increase in intracellular oxidative stress, leading to DNA damage. The activation of DNA damage repair mechanisms is crucial for preserving the integrity and function of neuronal cells [40,41]. The hypoxia-inducible factor signaling pathway is activated under hypoxic conditions, regulating the expression of a variety of genes, including those involved in angiogenesis, erythropoiesis, and cellular metabolism. These responses aid the body in adapting to hypoxic environments but may also be linked to the onset of cognitive impairment [42]. Changes in hypoxia-associated signaling pathways may impact neurotransmitter release and reuptake, thereby affecting cognitive function [43]. KEGG enrichment analysis revealed that the differential genes were enriched in signaling pathways such as neuroactive ligand–receptor interactions, glutamatergic synapses, and the cAMP signaling pathway. The cAMP signaling pathway is involved in the regulation of neural stem cell proliferation and differentiation, as well as the growth and regeneration of neuronal axons, and is closely related to the development of brain organoids. Proteins within this pathway, including protein kinase A (PKA), cAMP response element-binding protein (CREB), phosphodiesterase (PDE), and cAMP direct activation exchange protein (Epac), regulate the activity of the signaling pathway, thereby influencing neuronal excitability, synaptic transmission, and neuroplasticity, all of which are closely associated with cognitive dysfunction. The key differential gene PDE4C is enriched in the cAMP signaling pathway, suggesting that it is closely related to Dio’s protective effects against hypoxic brain organoid damage and may play a crucial role in preventing hypoxic cognitive impairment.

Phosphodiesterase 4 (PDE4) inhibition has been demonstrated to significantly ameliorate cognitive dysfunction in various neurodegenerative disorders [35]. PDE4 regulates cognitive function by modulating intracellular cyclic adenosine monophosphate (cAMP) levels, which subsequently influences cognitive performance. cAMP acts as a key second messenger involved in the regulation of synaptic plasticity and neuronal excitability. PDE4C, an isoform of the PDE4 family, can lead to decreased cAMP levels when its activity is elevated, thereby inhibiting protein kinase A (PKA) activity and ultimately affecting synaptic plasticity and memory formation [44]. Additionally, PDE4 inhibition has been shown to reduce microglial activation and inflammatory factor release, thereby attenuating neuroinflammation [45], findings that are consistent with our observations in the chronic plateau cognitive impairment study presented here. Our study revealed that Dio significantly inhibited the hypoxia-induced elevation in PDE4C expression and significantly restored the hypoxia-induced reduction in cAMP expression. Additionally, Dio exhibited high affinity for PDE4C. These results suggest that Dio is closely associated with the expression of PDE4C and cAMP and may serve as an important factor in alleviating hypoxia-induced brain damage and combating cognitive impairment.

## 4. Materials and Methods

### 4.1. Preparation of QJW Extract

ASR, AM, and PR were purchased from Beijing Tong Ren Tang (Beijing, China). PR was washed, sliced, and mixed with yellow rice wine (1:1 water-to-wine ratio) at a 5:1 ratio. The mixture was soaked at room temperature for 4 h, sterilized at 121 °C for 2.5 h, and then dried at 70 °C. ASR, AM, and PR were ground into fine powders and mixed in a 1:1:1 ratio. The mixture was refluxed with 10 times its volume of water for two 1 h cycles. The combined extracts were concentrated under reduced pressure at 65 °C using a Heidolph rotary evaporator (Beijing, China) and then freeze-dried into a powder using a Songyuan vacuum freeze-dryer (Beijing, China) for storage.

### 4.2. Grouping of Experimental Animals

#### 4.2.1. QJW Intervention in the Acute Plateau Model Mouse Experiment

Forty 7-week-old male C57BL/6J mice were acclimated for one week and then randomly assigned to the following groups: blank control (CON), acute plateau model (HHJ), and acute plateau model treated with low, medium, and high doses of QJW (Q-L, Q-M, Q-H). Mice from the HHJ, Q-L, Q-M, and Q-H groups were placed in a hypobaric hypoxic chamber to simulate exposure at an altitude of 7000 m for 72 h according to a previous study [46]. Mice in the CON group were housed in an environment with similar light, humidity, and temperature conditions but at an altitude of 100 m above sea level. Before being placed in a hypobaric hypoxic chamber, mice underwent continuous gavage of QJW for 11 days. The doses of QJW administered in the acute plateau model were determined based on the results of our team’s previous study [47]. Specifically, the Q-L, Q-M, and Q-H groups received 1.8 g/kg/day, 3.6 g/kg/day, and 7.2 g/kg/day of QJW, respectively. The lyophilized powder of QJW was prepared with saline, and the CON and HHJ groups were gavaged with equivalent volumes of saline. Body weights were measured every two days. After 72 h of hypobaric hypoxia exposure, mice were removed from the chamber for behavioral experiments and organ sampling. Following anesthesia, the experimental mice underwent surgical exposure of the brain. The brains of three mice were promptly excised and immersed in a 4% paraformaldehyde solution for fixation to maintain tissue integrity. The brains of the remaining mice were rapidly transferred to an ice-cold environment for dissection of the hippocampus and subsequent storage.

#### 4.2.2. QJW Intervention in Chronic Plateau Model Mice

Twenty-four 7-week-old male C57BL/6J mice were acclimated for one week. The optimal dose of QJW was determined by the previous experiments, and the mice were randomly assigned to the following groups: control (CON), chronic plateau model (HHM), and chronic plateau model with the QJW intervention (QJW). The HHM and QJW groups were placed in a hypobaric hypoxic chamber to simulate exposure at an altitude of 6000 m for 4 weeks. Mice in the CON group were housed in an environment with similar light, humidity, and temperature conditions but at an altitude of 100 m above sea level. Gavage administration of the drug commenced upon the mice’s entry into the hypobaric hypoxia chamber and continued until the conclusion of the experiment. With reference to prior studies, the QJW intervention was adjusted to a dosing regimen of once every two days at a dose of 7.2 g/kg [48]. The lyophilized powder of QJW was configured with saline, and the control and model groups were gavaged with equal doses of saline. Body weights were measured weekly, and mice were removed from the chamber for behavioral experiments as well as organ sampling after 4 weeks of hypobaric hypoxia exposure.

#### 4.2.3. Diosgenin (Dio) Intervention in Acute Plateau Model Mice

Twenty-four 7-week-old male C57BL/6J mice were acclimated for one week and randomly assigned to three groups: blank control (CON), acute plateau model (HHJ), and acute plateau model with the Dio intervention (Dio). The HHJ and Dio groups were placed in a hypobaric hypoxic chamber to simulate exposure at an altitude of 7000 m for 72 h. Mice in the CON group were housed in an environment with similar light, humidity, and temperature conditions (100 m above sea level). Diosgenin (HY-N0177) was purchased from MCE (Monmouth Junction, NJ, USA). The compound was dissolved in ethanol and then thoroughly mixed with corn oil to prepare the dosing solution. Prior to exposure in the hypobaric hypoxia chamber, mice in the acute plateau model received intraperitoneal injections of Dio at a dosage of 60 mg/kg/day for 11 consecutive days, referencing the optimal dose identified in the previous literature [49], while the control and model groups were given equivalent volumes of solvent. Body weights were measured every two days, and mice were removed from the chamber for behavioral experiments and organ sampling after 72 h of hypobaric hypoxia exposure.

### 4.3. Novel Object Recognition Test

The Novel Object Recognition Test (NOR) is a classical behavioral paradigm for assessing learning and cognitive memory abilities based on animal exploratory behavior. For acclimatization, mice were placed in an empty chamber for 5 min to eliminate the interference of environmental unfamiliarity on subsequent exploratory behaviors. In the learning stage, two identical objects (A and B) were placed symmetrically in the chamber, 10 cm away from the wall and fixed to avoid odor interference. Mice were released from the starting position (a point equidistant from the two objects) and their exploratory behaviors were recorded for 10 min using a video tracking system. In the test phase, one of the familiar objects (A or B) was replaced with a new object (C), maintaining positional symmetry. The system was then activated for a 5 min test. Immediately after each test, the objects were cleaned with 75% alcohol. Time spent exploring the old and new objects was recorded. The discrimination index (DR) was calculated as follows: DR = (time spent exploring the new object/(time spent exploring the new object + time spent exploring the old object)) × 100%. For the acute plateau model, both the QJW and Dio intervention groups underwent adaptation on day 11 after drug administration, learning on day 13, and testing on day 14 (Figure 1A and Figure 7A). For the chronic high-altitude model, the QJW intervention group underwent adaptation on day 28 after drug administration, learning on day 30, and testing on day 31 (Figure 2A).

### 4.4. Morris Water Maze Test

The Morris Water Maze Test (MWM) was used to assess rodents’ spatial learning and memory. The setup featured a circular pool with a 120 cm diameter, a 50 cm height, and an opaque black interior. A 10 cm diameter platform with a mesh surface was placed 1 cm below the water in the first quadrant’s center. A visual tracking system with an infrared camera and EthoVision XT 18 software recorded movement trajectories. The pool, filled with 25 ± 1 °C water mixed with milk powder for visibility, was divided into four quadrants with wall cues. Mice underwent five days of training, with four daily sessions from different quadrants. Each trial lasted 60 s; escape latency was recorded or capped at 60 s if the platform was not found. Intervals between sessions were ≥30 min. Post-training, a probe test removed the platform, and 24 h later, mice were released from a random quadrant for a 60 s free swim. Measurements including time in the target quadrant and swimming speed were analyzed to evaluate long-term memory. For the acute plateau model, both the QJW and Dio intervention groups underwent orientation training from day 6 to day 10 after drug administration, with testing conducted on day 14. For the chronic plateau model, the QJW intervention group underwent orientation training from day 32 to day 36 after drug administration, with testing conducted on day 37 (Figure 2A).

### 4.5. Hematoxylin and Eosin (HE) Staining

Brain tissues were fixed, embedded, and sectioned. Subsequently, the sections were subjected to xylene deparaffinization and gradient ethanol hydration. The sections were then stained with hematoxylin and eosin. The hippocampal tissue structure was examined at 400× magnification under a microscope. Three mice per group were selected, and four fields of view in the hippocampal region were photographed for each mouse. Image analysis software was employed to analyze the collected images and quantify the number of necrotic neurons in specific regions.

### 4.6. Nissl Staining

Paraffin sections were deparaffinized with xylene and hydrated using gradient ethanol. Nissl staining was performed and the chromogenic effect optimized through differential differentiation. Sections were then dehydrated with gradient ethanol, cleared with xylene, and sealed with neutral resin. The hippocampal tissue structure was examined at 400× magnification under a microscope. Three mice per group were selected, and four fields of view in the hippocampal region were photographed for each mouse. Image J 1.48v was used to analyze the collected images and quantify the number of Nissl-positive neurons in specific regions.

### 4.7. Immunofluorescence (IF) Detection

Coronal sections of the mouse hippocampus and sections of human brain organoids were prepared; primary antibodies were applied to cover the tissue, followed by overnight incubation in a humidified chamber at 4 °C. Subsequently, the corresponding secondary antibodies were added under light-avoidance conditions and incubated at 37 °C for 60 min. Unbound secondary antibodies were removed by washing with PBS, and sections were then sealed using an anti-fluorescence quenching mounting medium containing DAPI for nuclear staining. Sections were stored at 4° C in the dark. The antibodies used for IF included the following: anti-NeuN (1:5000, GB11138, Servicebio, Wuhan, China), anti-Iba-1 (1:500, GB113502, Servicebio), anti-p-VIM (1:300, D076-3, MBL International, Woburn, MA, USA), anti-MAP2 (1:250, 17490-1-AP, Proteintech, Wuhan, China), anti-PAX6 (1:300, 20932-1-AP, Proteintech), anti-TUJ1 (1:300, MMS-435P, BioLegend, San Diego, CA, USA), anti-SOX2 (1:200, 11064-1-AP, Proteintech), anti-PDE4C (1:300, PA5-106624, Invitrogen, Carlsbad, CA, USA), anti-HRP-labeled goat anti-rabbit secondary antibody (1:500, GB23303, Servicebio), and CY3-labeled goat anti-rabbit secondary antibody (1:300, GB21303, Proteintech). Non-specific binding was blocked with 10% goat serum prior to primary antibody application. At the end of the staining process, the hippocampal and brain organoid tissues were examined using a confocal laser scanning microscope at magnifications of 400× and 40×. For each experimental group, three mice were selected, and four fluorescent images of the hippocampal region were captured per mouse. Similarly, three brain organoids per group were selected, and three fluorescent images were captured per organoid. Image analysis software was employed to analyze the collected images and quantify the fluorescence intensity of proteins in specific regions.

### 4.8. Ultrastructural Observation of Hippocampal Tissue

Mouse hippocampi were collected and immediately fixed in fresh pre-cooled 2.5% glutaraldehyde solution at 4 °C overnight. Subsequently, the samples were rinsed with 0.1 M phosphate buffer for 10 min three times. Next, samples were sequentially dehydrated with gradient ethanol, followed by infiltration and embedding with epoxy resin. Finally, 70 nm thick slices were prepared using an ultrathin slicer and double-stained with uranyl acetate and lead citrate, and hippocampal synaptic structures were observed using transmission electron microscopy.

### 4.9. Multifactor Assay and Enzyme-Linked Immunosorbent Assay (ELISA)

A cytokine and chemokine kit from Leitz Biotechnology Co., Ltd. (Beijing, China). was used to detect the levels of IL-1β, IL-6, and TNF-α in mouse serum. The antigen standard was prepared and then diluted. Magnetic beads were added to the diluted standard and vortexed for 30 s, and 50 μL of the bead mixture was added to each well. After removing the liquid, 25 μL of the serum samples was added to the wells. The plate was sealed and incubated with shaking at room temperature for 120 min. The beads were then washed three times. Following this, 25 μL of a detection antibody mixture was added to the wells, which were then sealed and incubated with shaking for 30 min. The beads were washed three times again before was adding streptavidin. The beads were incubated with shaking for another 30 min and washed three times. Finally, 120 μL of buffer was added to resuspend the beads, which were then sealed and shaken for 5 min before being assayed on the machine to collect data. The expression levels of PDE4C and cAMP in brain organoids were determined using an enzyme-linked immunosorbent assay (ELISA, Wuxi, China) kit. The organoids were homogenized thoroughly using a homogenizer and then centrifuged at 2–8 °C for 20 min at 2000–3000 rpm. The supernatant was collected and the prepared samples and standards added. The mixture was incubated at 37 °C for 30 min. After washing the plate five times, a second incubation was performed at 37 °C for 30 min. Following another five washes, chromogenic substrates A and B were added and the plate was incubated at 37 °C for 10 min. The reaction was terminated by adding the stop solution. The absorbance of each well was measured sequentially at 450 nm using a microplate reader within 15 min.

### 4.10. UPLC-MS/MS Detection of QJW and Screening of Potential Active Ingredients

QJW lyophilized powder was dissolved in 1 mL of methanol–water (4:1, *v*/*v*) containing 10 μg/mL internal standard. After vortexing for 30 s and sonication in an ice–water bath for 1 h, the sample was stored at −40 °C for 1 h to induce precipitation. Then, centrifugation was performed at 12,000 rpm for 15 min at 4 °C, and the supernatant (500 μL) was filtered through a 0.22 μm membrane for analysis. UHPLC was performed on a Waters UPLC BEH C18 column (2.1 mm × 100 mm, 1.7 μm) at 55 °C with a 5 μL injection volume and a 0.5 mL/min flow rate. Mobile phases were 0.1% formic acid in water (A) and 0.1% formic acid in acetonitrile (B). Gradient elution: 85% A to 25% A over 0–11 min, 2% A at 11–14 min, and re-equilibration to 85% A by 16 min. The UHPLC was coupled to a Q Exactive Focus mass spectrometer with ESI in an alternating positive/negative mode. Parameters: spray voltage: ±4.0/3.6 kV; sheath gas: 45 Arb; auxiliary gas: 15 Arb; capillary temperature: 400 °C. Full MS scan (*m*/*z* 100–1500, 70,000 resolution), with top 3 ions fragmented (DDA mode, MS/MS resolution 17,500, NCE steps 15/30/45). Raw data were processed using XCMS, and components were identified via a secondary MS database, retention time, and fragment ion matching. A chemical composition database was established. Using the TCMSP database, QJW ingredients that could cross the blood–brain barrier were selected and cross-referenced with the in-house database. Ingredients were further screened based on oral bioavailability and druglikeness for potential cognitive improvement effects.

### 4.11. Brain Organoid Culture, Characterization, and Intervention

Brain organoid models were generated using H1 human embryonic stem cells and the STEMdiff™ Brain Organoid Kit (STEMCELL Technologies, Vancouver, Canada). The protocol involved multi-stage induction: H1 cells were first expanded in Matrigel-coated 6-well plates with the mTeSR1 medium until 70–80% confluence, then dissociated into single cells and seeded into low-adhesion 96-well plates at 9 × 10^3^ cells per well. After centrifugation (200× *g*, 5 min), embryoid bodies (EBs) were formed in the EB medium (mTeSR1, STEMdiff™ Basal Medium 1, Supplement A, and Y-27632). From day 1 to day 4, the medium was refreshed every other day to stabilize EB structure. On day 5, cells were transferred to low-adhesion 24-well plates and cultured in Neuroinduction Medium (Basal Medium 1 and Supplement B) for 48 h to initiate neural differentiation. On day 7, EBs were embedded in Matrigel drops and cultured in the expansion medium (Basal Medium 2, Supplements C and D) to promote neuroepithelial growth. From day 10, maturation medium (Basal Medium 2 and Supplement E) was used, and cultures were shaken at 60 rpm until day 40 to facilitate cortical layering and synaptic network formation. Cultures were maintained at 37 °C with 5% CO_2_, and organoid diameters were monitored weekly. After maturation, immunofluorescence was used to detect markers such as PAX6, SOX2, TUJ1, MAP2, p-VIM, and NeuN. Mature organoids were randomized into three groups: blank control (P), hypoxic model (H), and hypoxic model with the diosgenin (Dio) intervention (H_Dio). Groups H and H_Dio were exposed to 1% O_2_ for 48 h in a hypoxic incubator [36,50], while the H_Dio group received 5 μM Dio concurrently. At the end of the experiment, part of the organoids was snap-frozen in liquid nitrogen for storage at −80 °C, and the remaining part was fixed in paraformaldehyde for embedding and sectioning.

### 4.12. Brain Organoid Transcriptome Sequencing

Brain organoids from each group were snap-frozen in liquid nitrogen for sequencing. Total RNA was extracted and used to construct sequencing libraries. Oligo(dT)-modified magnetic beads specifically captured eukaryotic mRNAs to exclude non-target RNAs. After quality control, mRNAs were fragmented chemically and reverse transcription was initiated with random hexamer primers to synthesize single-stranded cDNA, followed by double-stranded cDNA synthesis using DNA polymerase I and RNase H. The cDNA was purified via magnetic bead adsorption to remove enzyme residues and by-products. Purified cDNA underwent end repair, adenine overhang addition, and adapter ligation. Size selection and PCR amplification were performed to generate standardized libraries, which were sequenced after quality control. Raw data were processed with fastp to remove adapters and low-quality reads [51], and clean reads were aligned to the hg38 genome using Salmon [52]. Differential expression analysis was conducted with DESeq2 [53], identifying DEGs with |log_2_(fold change)| > 1 and adjusted *p* < 0.05. These DEGs were classified as up-regulated or down-regulated and analyzed for biological processes and KEGG pathways using clusterProfiler to explore their functional roles [54].

### 4.13. Molecular Docking

The three-dimensional structure of PDE4C (in the PDB format) was obtained from the PDB database (http://www.rcsb.org) and saved in the PDB format after removing water molecules and small-molecule ligands using the software Pymol (https://pymol.org/). Subsequently, the processed structures were hydrogenated using the software Autodock (https://autodock.scripps.edu/) and converted to the PDBQT format for subsequent use. The two-dimensional structure of Dio (in the SDF format) was retrieved from the PubChem database (https://pubchem.ncbi.nlm.nih.gov), optimized for minimum free energy using the software ChemBio3D Ultra 14.0.0.117, and temporarily stored in the MOL2 format. It was then converted into the PDBQT format using the software Autodock. Finally, molecular docking experiments were conducted using the software Autodock and the minimum binding energy was recorded.

### 4.14. Statistical Analysis

All data are presented as the mean ± standard deviation (Mean ± SD). Statistical analysis was conducted using GraphPad Prism version 9.5. Intergroup comparisons were made using one-way ANOVA, followed by Tukey HSD post hoc testing to ascertain specific pairwise differences. A *p*-value less than 0.05 was considered statistically significant.

## 5. Conclusions

In conclusion, this study demonstrated that QJW effectively prevented and treated hypobaric-hypoxia-induced neuronal and synaptic damage in the hippocampal tissue, inhibited microglial activation, reduced systemic inflammation, and mitigated cognitive impairment in plateau model mice. Dio alleviates hypoxia-induced brain damage by modulating the cAMP signaling pathway and regulating the expression of key genes, such as PDE4C, thereby preventing and controlling cognitive impairment.

## Data Availability

The original contributions presented in this study are included in the article. Further inquiries can be directed to the corresponding author.

## Supplementary Materials

The following supporting information can be downloaded at: https://www.mdpi.com/article/10.3390/ph18050738/s1, Figure S1: Analysis of Differentially Expressed Genes in Brain Organoids and H&E Staining of Mouse Liver. (A, B) GO and KEGG enrichment analysis of differentially expressed genes (DEGs). (C) Average fold change of DEGs among groups. (D) Representative H&E staining images of mouse liver sections from CON, HHM, and QJW groups. Supplementary Table S1: 12 Intersecting Components Between QJW and BBB.
