# Supplementary material for: Neuroprotective Effects of Qi Jing Wan and Its Active Ingredient Diosgenin Against Cognitive Impairment in Plateau Hypoxia
Source: Pharmaceuticals (Basel). 2025 May 17;18(5):738. doi: 10.3390/ph18050738 (PMC12114856; doi:10.3390/ph18050738)
Supplement: Supplementary file 1 [file pharmaceuticals-18-00738-s001.zip › Attached table.pdf]

Supplementary tables

Supplementary Table S1 12 Intersecting Components Between QJW and BBB

NO	Molecule ID	NameEN	InChIKey	MW	OB (%)	BBB	DL	Source
1	MOL000546	Diosgenin	WQLVFSAGQJTQCK-VKROHFNGSA-N	414.69	80.88	0.27	0.81	PR
2	MOL000392	Formononetin	HKQYGTCOTHHOMP-UHFFFAOYSA-N	268.28	69.67	0.02	0.21	AM
3	MOL008251	Sedanolide	UPJFTVFLSIQQAV-GXSJLCMTSA-N	194.3	62.46	1.4	0.07	ASR
4	MOL000414	Caffeate	QAIPRVGONGVQAS-RQOWECAXSA-N	180.17	54.97	0.11	0.05	AM
5	MOL000635	Vanillin	MWOOGOJBHIARFG-UHFFFAOYSA-N	152.16	52	0.41	0.03	ASR
6	MOL000421	Nicotinic acid	PVNIIMVLHYAWGP-UHFFFAOYSA-N	123.12	47.65	0.21	0.02	AM
7	MOL005500	Linolenic acid	DTOSIQBPPRVQHS-IUQGRGSQSA-N	278.48	45.01	0.84	0.15	AM
8	MOL000449	Stigmasterol	HCXVJBMSMIARIN-PHZDYDNGSA-N	412.77	43.83	1	0.76	ASR
9	MOL000358	Beta-sitosterol	KZJWDPNRJALLNS-VJSFXXLFSA-N	414.79	36.91	0.99	0.75	ASR、PR
10	MOL001801	Salicylic acid	YGSDEFSMJLZEOE-UHFFFAOYSA-N	138.13	32.13	0.63	0.03	PR
11	MOL000069	Palmitic acid	IPCSVZSSVZVIGE-UHFFFAOYSA-N	256.48	19.3	1	0.1	AM
12	MOL000394	Choline	GDPPXFUBIJJIKR-UHFFFAOYSA-N	104.2	0.47	0.64	0.01	AM
