# Supplementary figures and images for: Neuroprotective Effects of Qi Jing Wan and Its Active Ingredient Diosgenin Against Cognitive Impairment in Plateau Hypoxia
Source: Pharmaceuticals (Basel). 2025 May 17;18(5):738. doi: 10.3390/ph18050738 (PMC12114856; doi:10.3390/ph18050738)

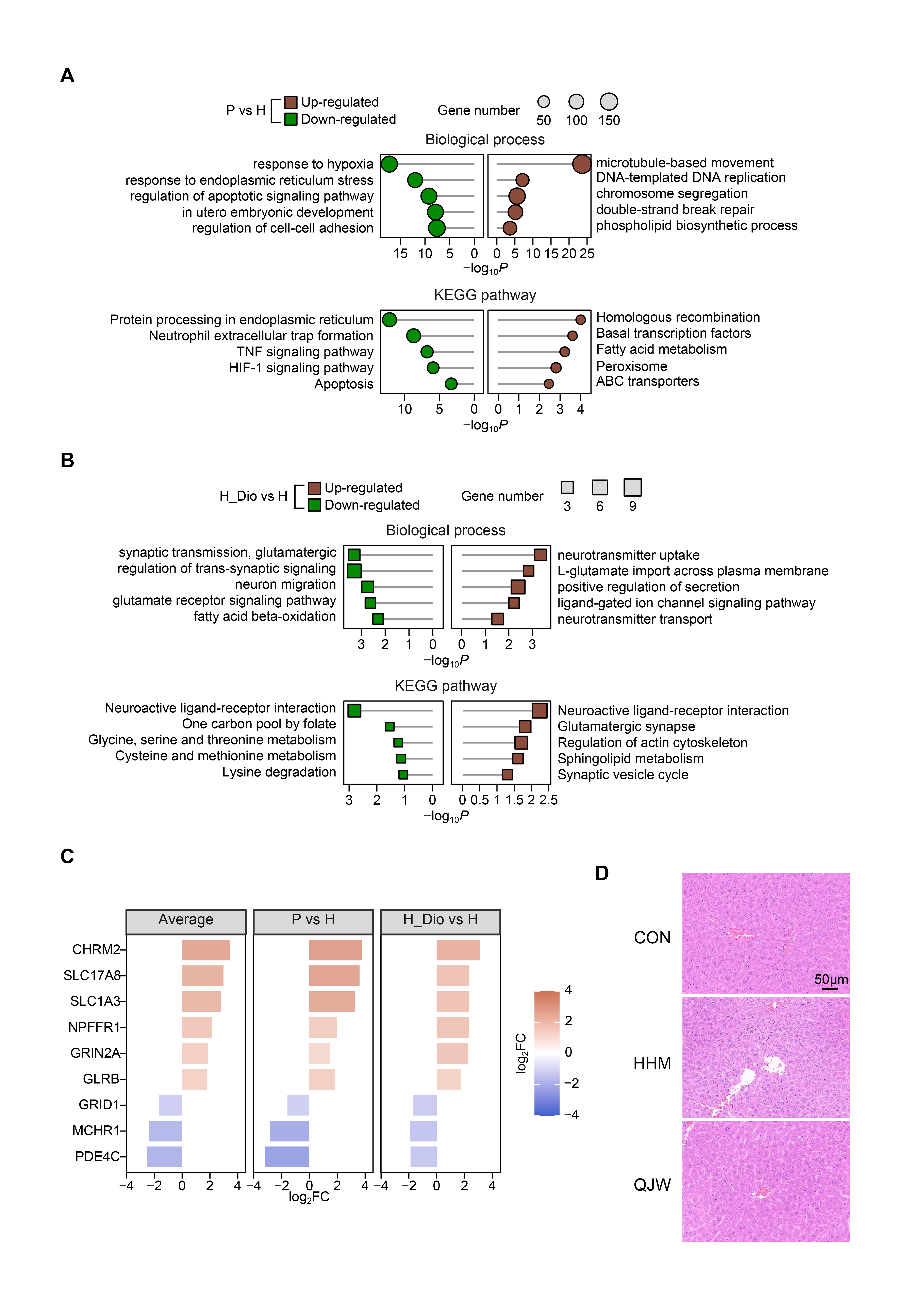

Supplement: Supplementary file 1 [file pharmaceuticals-18-00738-s001.zip › figure S1.jpg]
